# Wearable Antennas for Sensor Networks and IoT Applications: Evaluation of SAR and Biological Effects

**DOI:** 10.3390/s22145139

**Published:** 2022-07-08

**Authors:** Nikolay Todorov Atanasov, Gabriela Lachezarova Atanasova, Boyana Angelova, Momchil Paunov, Maria Gurmanova, Margarita Kouzmanova

**Affiliations:** 1 Department of Communication and Computer Engineering, South-West University “Neofit Rilski”, 2700 Blagoevgrad, Bulgaria; gatanasova@swu.bg; 2 Department of Biophysics and Radiobiology, Sofia University “St. Kliment Ohridski”, 1164 Sofia, Bulgaria; angelova_bd@uni-sofia.bg (B.A.); mokavey@abv.bg (M.P.); m.gurmanova@uni-sofia.bg (M.G.); mkouzmanova@uni-sofia.bg (M.K.)

**Keywords:** wearable antennas, fully textile antenna, antenna for sensor applications, SAR, biological effects, EMF, reactive near-field, erythrocyte membrane

## Abstract

In recent years, there has been a rapid development in the wearable industry. The growing number of wearables has led to the demand for new lightweight, flexible wearable antennas. In order to be applicable in IoT wearable devices, the antennas must meet certain electrical, mechanical, manufacturing, and safety requirements (e.g., specific absorption rate (SAR) below worldwide limits). However, the assessment of SAR does not provide information on the mechanisms of interaction between low-intensity electromagnetic fields emitted by wearable antennas and the human body. In this paper, we presented a detailed investigation of the SAR induced in erythrocyte suspensions from a fully textile wearable antenna at realistic (net input power 6.3 mW) and conservative (net input power 450 mW) conditions at 2.41 GHz, as well as results from *in vitro* experiments on the stability of human erythrocyte membranes at both exposure conditions. The detailed investigation showed that the 1 g average SARs were 0.5758 W/kg and 41.13 W/kg, respectively. Results from the *in vitro* experiments demonstrated that the short-term (20 min) irradiation of erythrocyte membranes in the reactive near-field of the wearable antenna at 6.3 mW input power had a stabilizing effect. Long-term exposure (120 min) had a destabilizing effect on the erythrocyte membrane.

## 1. Introduction

In recent years, there have been rapid developments in the wearable industry. The forecast by Statista shows an approximately three-fold increase in the number of connected wearable devices worldwide over six years (from 325 million in 2016 to 1105 million in 2022) [1]. Additionally, applications for wearable devices and wireless sensor networks for remote patient monitoring experienced an acceleration in growth during the COVID-19 pandemic. The growing number of wearables has led to demand for lightweight, flexible, fully textile, or optically transparent wearable antennas which can be integrated into clothing or accessories. 

Depending on the wearable device application (e.g., health care, sports, advertising), antennas can be directly mounted on a human arm, forearm, back, chest, leg, or neck or integrated into a garment or accessory. Due to the presence of the human body, wearable antenna design is very complicated; antennas must meet electrical (bandwidth, radiation efficiency, gain), mechanical (low profile, flexible, lightweight), manufacturing (low cost, simple structure), and safety requirements (specific absorption rate (SAR) below the worldwide standard limits) [2,3] to be applicable in Internet of Things (IoT) wearable devices. Furthermore, in order to estimate the applicability of antennas for body-worn wireless devices, the antenna parameters and characteristics need to be studied both in free space and on the human body [4]. Several types of flexible wearable antennas for embedding into garments or accessories have been presented that fully or partially meet the design requirements: patch antennas [5,6,7,8,9,10], textile slotted waveguide antennas [11], monopole textile antennas [12,13], and fully-textile loop antennas [14,15]. The effects of the human body on antenna parameters and characteristics has been numerically investigated by employing homogenous (cylindrical [11], rectangular [11,14], forearm [5], full-scale human body model [7]) and multilayer (cylindrical [6], rectangular [15]) human body models.

The assessment of the absorption of electromagnetic energy by human tissues is an important topic to be considered. So are the health risks that could arise as a consequence of exposure to electromagnetic fields (EMFs) produced by a wearable antenna placed on or very close to the human body (24 h/7 days a week) [3,16]. In the frequency range of 100 kHz to 6 GHz, the SAR is defined as an indication of how much electromagnetic energy is absorbed by human tissues [17,18]. The SAR limits for local exposures in the considered frequency range were established in recent ICNIRP guidelines and IEEE C95.1-2019 standard [17,19]. Several previously reported works on fully textile and optically transparent wearable antennas showed that the simulated peak 1 g and 10 g average SAR generated from the antennas in a homogeneous flat phantom [14], in the chest, upper arm, and the wrist of the HUGO human body model [7], in a multilayer cylindrical human body model [6] and in a forearm phantom [5] varied from 0.22 to 0.533 W/kg (SAR 1 g) and from 0.148 to 0.695 W/kg (SAR 10 g), at 2.45 GHz at a net input power of 100 mW.

Moreover, the assessment of SAR does not provide information on the mechanisms of interaction between low-intensity EMFs emitted by wearable antennas and the human body. Epidemiological, *in vivo*, and *in vitro* experiments have been employed to study possible health risks of human exposure to EMFs [17,20,21]. In the last three decades, numerous studies on the biological effects associated with EMFs at 2.45 GHz have been carried out. Endocrine and hematological changes have been observed in mice [22]. Studies have shown that 2.45 GHz irradiation has an adverse effect on the erythrocyte membrane, causing changes in its osmotic resistance by increasing the rate of hemolysis [23]. Kiel and Erwin [24] observed significantly decreased hemolysis of human erythrocytes exposed *in vitro* to microwave EMF (2.45 GHz, pulsed, 5 mW/cm^2^ power flux density, 20 min exposure, SAR 400 µW/g) at 42 °C, but there was no field effect at 37 °C or 48 °C. The biological effects of EMFs are considered to have two main components: thermal and non-thermal levels of action [25,26,27]. Kim et al. [28] investigated the effects of 2.45 GHz EMF with different SARs (below and above 500 W/kg). They found that an *in vitro* hemolytic process induced by strong (SAR above 500 W/kg) EMFs was caused by thermal denaturation of protein domains of the erythrocyte membrane skeleton and did not differ from the hemolytic process caused by heat at the same values of temperature and heating rate. Prolonged exposure to EMF with a relatively low SAR (below 500 W/kg) can induce hemolysis without integral heating of a cell suspension above 37–40 °C.

The effects of long-term exposure of human blood to 2.45 GHz (continuous wave (CW), 60 h exposure, 84 h kinetic measurements, 10 mW/cm^2^ power flux density) were studied in [29]. A significant increase in hemoglobin loss by irradiated erythrocytes was observed, as well as a strong dependence of the rate of the increase of hemoglobin loss on the initial level of the spontaneous hemolysis. It was found that, at low power densities, the hemolysis degree increased quasi-linearly with the exposure time, while, at higher power density (5 mW/cm^2^), this tendency was reversed after the first 10 h of the irradiation.

In most of these studies, erythrocyte suspensions were exposed to different types of incident fields (mostly far-field conditions) at one or multiple exposure levels which were significantly higher than those emitted by the wearable antennas. A portion of the conducted studies suffered from poor dosimetry [22,29]; for example, there was no information on the values of the SAR or SAR distribution in the suspension. Consequently, in order to understand the possible adverse human health effects of such electromagnetic fields, the mechanism of interaction between EMFs emitted by wearable antennas and the biological systems must be studied.

In this paper, we presented a detailed investigation of SAR induced in erythrocyte suspensions from a fully textile wearable antenna at realistic (net input power 6.3 mW) and conservative (net input power 450 mW) conditions at 2.41 GHz and results from *in vitro* experiments on the stability of human erythrocyte membranes at both exposure conditions. Section 2 describes models, methods, and experimental procedures used in numerical and biological experiments. Section 3 presents the results of a detailed numerical study of the SAR in erythrocyte suspensions at two input powers of the wearable antenna. The results of *in vitro* experiments on the stability of human erythrocyte membranes at different duration and exposure levels are also given in Section 3.

## 2. Methods, Models, and Experimental Procedures

### 2.1. Methods and Models

#### 2.1.1. Numerical Method and Models

The finite-difference time-domain (FDTD) method was used to perform a numerical analysis of the propagation of electromagnetic waves and SAR distributions within the erythrocyte suspensions and to provide the vectors of the electric and magnetic fields of the wearable antenna. The method was chosen because it is one of the most popular tools to study electromagnetic wave interactions with biological systems [17,30]. 

A cubical mesh of 0.5 mm × 0.5 mm × 0.5 mm was used for the numerical models of the wearable antenna, erythrocyte suspension, flat phantom, and free space. Two types of sources (Gaussian (pulse width of 32-time steps) and sinusoidal (at 2.41 GHz) excitations) were used in the numerical simulations. The duration of each simulation was 40,000-time steps.

The electromagnetic properties of the materials involved in the wearable antenna geometry, erythrocyte suspension, plastic cuvette, and flat phantom are shown in Table 1. The detailed geometrical dimensions of the wearable antenna are presented in [14].

#### 2.1.2. Experimental Method

Hemolysis of the erythrocytes was monitored by the absorbance of the supernatant at 413 nm in a spectrophotometer. We chose the 413 nm wavelength because hemoglobin has a maximum absorption at this wavelength. Hemoglobin concentrations were calculated using the equation described in [31].

### 2.2. Experimental Procedures

#### 2.2.1. Erythrocytes, Isolation, and Preparation of the Cell Suspensions

Blood from healthy donors (blood group 0 or A, Rh+ or Rh–) was obtained from the National Center for Transfusion Hematology (Sofia, Bulgaria) and was stored at 4 °C until used. The experiments were performed with 1, 2, and 3-week of blood storage.

Erythrocytes (red blood cells—RBCs) were washed three times in 0.155 M NaCl (blood was centrifuged one time at 1500 rpm for 5 min to remove the blood plasma and other components after that blood was centrifuged two times at 2000 rpm for 10 min). The obtained cell suspensions were diluted to a final cell concentration of 40% hematocrit (Ht) with phosphate-buffered saline (PBS, pH 7.4).

#### 2.2.2. Experimental Groups

The erythrocyte suspensions were divided into four groups: control (not exposed, also called sham-exposed), EMF exposed (at 2.41 GHz), conventional heat exposed (at 38 °C), and control left at room temperature (at 24 °C). 

The erythrocyte suspensions in the control group were placed in the same environmental conditions as those of EMF exposed samples but in absence of EMF and heat exposure. 

The erythrocyte suspensions in the conventional heat exposed group were incubated in a 38 °C-tempered water bath for 60, 120, 180, and 240 min (corresponding to the EMF exposure duration). This group was used to assess the effects of conventional (non-EMF induced) heating on erythrocytes. Moreover, erythrocyte suspensions left at room temperature (24 °C) were considered controls at 24 °C.

For the four groups of erythrocyte suspensions, the same type of cuvette was used, filled with 2 mL of final cell concentration 40% hematocrit.

Experiments were run in parallel, with control (not exposed), EMF (at 2.41 GHz) and conventional heat exposed (at 38 °C) erythrocyte suspensions, at durations of exposure of 20 min or 120 min.

#### 2.2.3. Exposure Setups and Conditions

Two experimental setups were designed and implemented to study the impact of EMF from wearable antennas on RBCs membranes.

**Figure 1:**
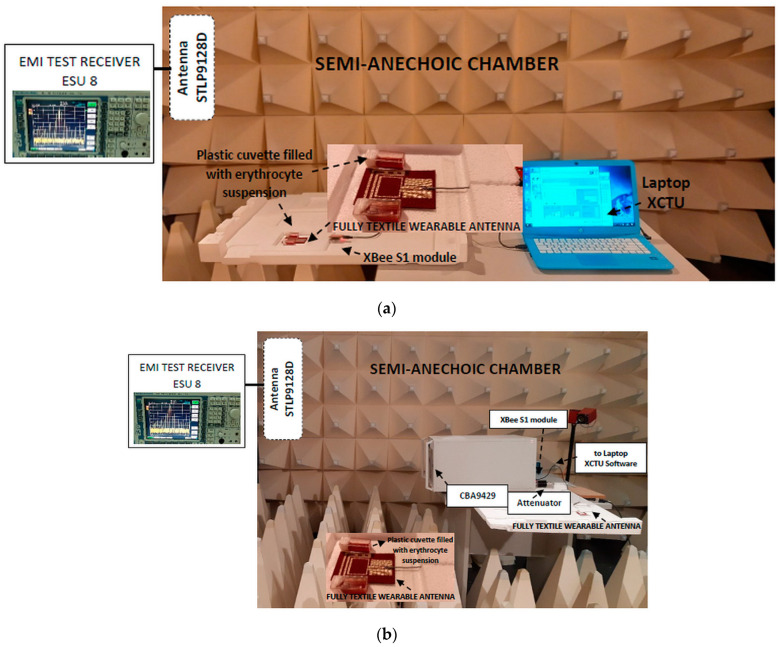
Overview of the exposure setups for investigation of the biological responses at: (**a**) realistic exposure conditions (net input power 6.3 mW) relevant to actual human exposure conditions when the antenna is placed on the human body; (**b**) conservative exposure conditions (net input power 450 mW) at sufficiently high exposure levels.

The first exposure setup is shown in Figure 1a. It was developed to study the stability of human erythrocyte membranes to electromagnetic fields from a fully textile wearable antenna at realistic exposure conditions (relevant to actual human exposure conditions when the antenna is placed on the human body). 

**Figure 2:**
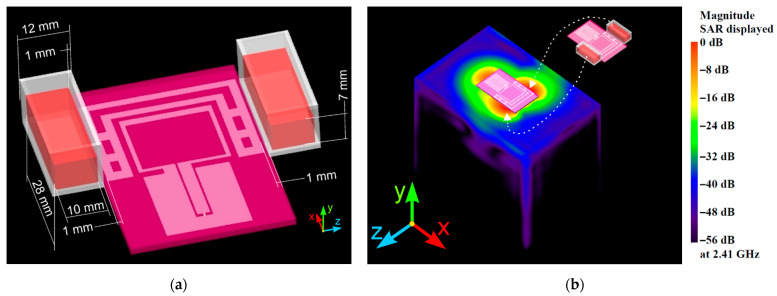
Numerical models of (**a**) wearable antenna, cuvettes, and erythrocyte suspension, and (**b**) flat homogeneous phantom and SAR distribution on the surface of the phantom induced from the wearable textile antenna, as incorporated in the FDTD model.

Geometry and details about the wearable antenna numerical model, fabrication, and testing (in free space and on a human body phantom) of the wearable antenna prototype with a polyester substrate used to expose the RBCs can be found in our previous work [14]. Details about wearable antenna parameters (such as VSWR, efficiency, gain, etc.) for two scenarios ((1) antenna in free space and (2) antenna with two cuvettes filled with 2 mL erythrocyte suspensions) were presented in Section 3. The distance between the wearable antenna radiating elements and two cuvettes filled with RBCs suspension remained constant (distance 1 mm, see Figure 2a).

In the first exposure setup, the wearable antenna with a polyester substrate from [14] was fed via a coaxial cable from an XBee S1 802.15.4 radio frequency module (Digi International Inc., Thief River Falls, MN, USA) controlled by a computer to emit a Zigbee-like signal at 2.41 GHz, with output power of 6.3 mW, as shown in Figure 1a. The minimum time to wait before sending a new packet was set to 1 ms. An EMI receiver (located outside the semi-anechoic chamber) and antenna (STLP99128D) were used to observe the signal emitted by the wearable antenna during the exposure. We chose the 2.41 GHz frequency because it corresponded to the radio channel 12 in a Zigbee network. This channel is usually used when the ZigBee Coordinator forms a network in ISM 2.4 band. In practice, the ZigBee Coordinator analyzes the signal spectrum to detect energy levels on each channel. After that, the coordinator removes channels with excessive energy levels from its list of potential channels to start on.

In order to evaluate the biological response at sufficiently high exposure levels, a new experimental setup, shown in Figure 1b, was developed. The Zigbee-like signal at 2.41 GHz was generated with the XBee S1 802.15.4 radio frequency module, which was connected to a microwave amplifier (CBA9429) and the wearable antenna was fed with 450 mW input power via a coaxial cable from the microwave amplifier (through a 6 dB attenuator). The minimum time to wait before sending a new packet was set to 1 ms. 

All exposures were performed in a semi-anechoic chamber.

The cuvettes were placed in the positions of the maximum SAR because the exposure took place in the reactive near-field of the wearable antenna. The location of the maximum SAR was determined by FDTD numerical simulations when the antenna was mounted on a flat phantom. The results are presented in Figure 2b. From the SAR distribution on the surface of the phantom, we observed three areas in which the SAR values were close to the maximum SAR value (varying in range from 0 dB to −16 dB). The first area was under the coplanar waveguide (CPW). Moreover, two areas with high SAR values occurred on the phantom surface near the radiating elements along the x-axis. Cuvettes with erythrocyte suspensions were placed in these two areas to be investigated under the worst-case scenario (maximum SAR conditions).

The detailed SAR values and SAR distributions at the location of the cell suspensions and inside them were presented in Section 3.

#### 2.2.4. Duration of Exposure

The erythrocyte suspensions were exposed (or sham-exposed) for 20 min, 60 min, 120 min, 180 min, or 240 min in order to investigate the biological response at short- and long-term exposures.

**Figure 3:**
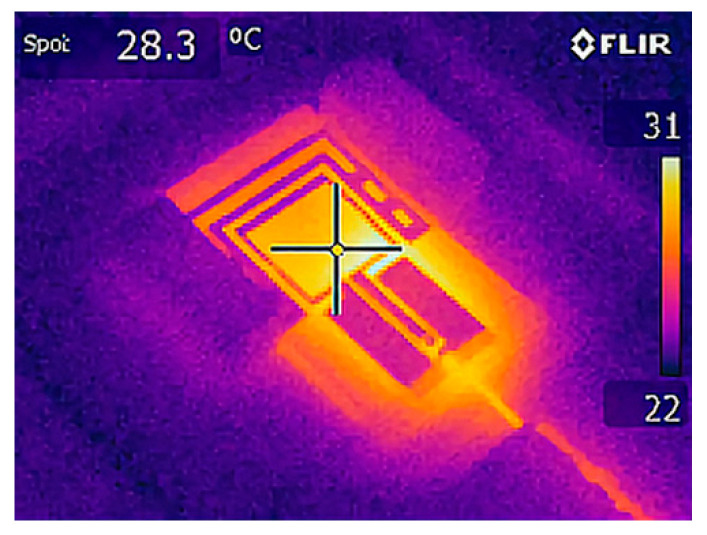
Thermographic image of the wearable antenna and the two cuvettes with erythrocyte suspensions immediately after EMF exposure.

The temperature in the wearable antenna substrate and cell suspensions was measured before and immediately after exposure by an infrared camera (FLIR E5). A thermographic image, taken immediately after exposure, is shown in Figure 3. Temperature distribution on the surface of the cuvettes with erythrocyte suspensions was determined by FLIR Thermal Studio Suite.

### 2.3. Statistical Analysis

Data were expressed as the mean ± SEM. The results of the released hemoglobin by control (sham-exposed) and EMF-exposed groups were normalized to the control group to avoid the effects of external factors, such as blood storage time, different donors, and blood groups. For this reason, the values of the released by control group hemoglobin were always 1 (normalized to themselves) without standard errors (standard errors were zero).

## 3. Results and Discussion

### 3.1. Wearable Antenna Performance

To evaluate how the cuvettes filled with erythrocyte suspensions could impact the wearable antenna parameters and characteristics, we estimated (numerically and experimentally) the antenna performance in two scenarios: (1) antenna alone in free space and (2) antenna with two cuvettes filled with RBCs suspension. 

**Figure 4:**
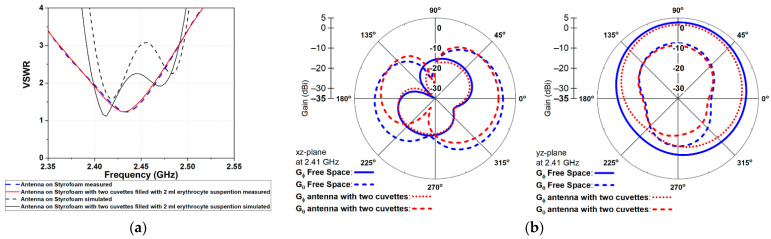
Antenna performance: (**a**) VSWR and (**b**) 2D radiation patterns in xz- and yz-plane.

The simulated and measured VSWR is shown in Figure 4a. From the measurements, we observed that the wearable antenna showed a very stable VSWR in the target ISM 2.4 GHz band when the two cuvettes filled with RBCs suspension were located at a distance of 1 mm from the antenna radiating elements. The resonant frequency and bandwidth remained almost the same when compared with those of the wearable antenna in free space. Moreover, a slight difference existed between measured and simulated results. It was noted that VSWR was slightly reduced when two cuvettes were placed close to the antenna radiating elements. 

The far-field radiation patterns in the xz- and yz-plane of the wearable antenna alone in free space and the wearable antenna with two cuvettes filled with erythrocyte suspension are shown in Figure 4b. As shown, the two cuvettes filled with erythrocyte suspension slightly decreased the gain components of the antenna compared to the free space. A difference of 2.19 dB and 1.14 dB in the direction of maximum radiation was observed for xz- and yz-plane, respectively.

Finally, a comparison of the simulated antenna parameters (input impedance, VSWR, radiation efficiency, and system efficiency) at 2.41 GHz in two scenarios is given in Table 2. As shown in the results, we observed that the wearable antenna with two cuvettes exhibited a better impedance matching compared with the antenna in free space. Moreover, the total radiation and system efficiency were reduced by 16% and 11%, respectively, compared to the free space. The reduction in radiation efficiency could be attributed to the fact that part of the radiated energy was absorbed in RBCs suspensions. As shown, the dissipated power increased by 17% compared to the free space scenario.

From the results, we were able to conclude that the performance of the wearable antenna was well maintained when two cuvettes with erythrocyte suspension were placed close (1 mm) to antenna radiating elements.

### 3.2. Numerical Estimation of the Induced Specific Absorption Rate

A detailed numerical SAR estimation was carried out, based on FDTD numerical simulations. The geometry of the wearable antenna and cuvettes with cell suspension, as incorporated in the FDTD model, is shown in Figure 2. The electromagnetic properties of all materials involved in the numerical models of the wearable antenna geometry, plastic cuvette, and erythrocyte suspension are presented in Table 1. In all simulations, the antenna radiating elements were located at a distance of 1.00 mm from the walls of the cuvettes, as shown in Figure 2.

First, the peak spatial-average SAR in 1 g of the numerical model of the erythrocyte suspension at two different input powers (6.3 mW and 450 mW) at 2.41 GHz was estimated. The results are provided in Table 3. As can be seen, at a net input power of 6.3 mW, the peak 1 g spatial-average SAR produced in the numerical model of the erythrocyte suspension was 0.5758 W/kg. This peak 1 g spatial-average SAR value was much lower than the maximum allowed value of 1.6 W/kg [32]. Moreover, at a net input power of 450 mW (4.5 times higher than usually allowed for wearable devices’ maximum power of 100 mW), the peak 1 g spatial-average SAR produced in the numerical model of erythrocyte suspension was 41.13 W/kg. Comparing the values of 1 g spatial-average SAR, it was noted that, at 450 mW, the wearable antenna produced nearly 70 times higher SAR, compared to the input power of 6.3 mW. The SAR averaged over the entire mass of the numerical model of the erythrocyte suspension (WSA SAR) was also estimated. The results are presented in Table 3.

**Figure 5:**
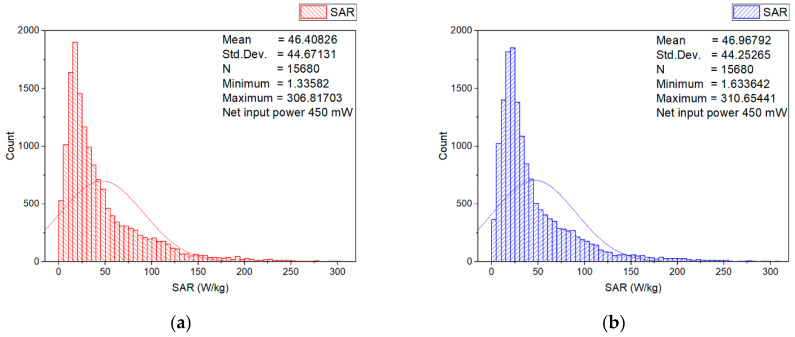
Histograms and descriptive statistics of SAR distribution for the erythrocyte suspensions at net input power of 450 mW in: (**a**) left and (**b**) right cuvette.

**Figure 6:**
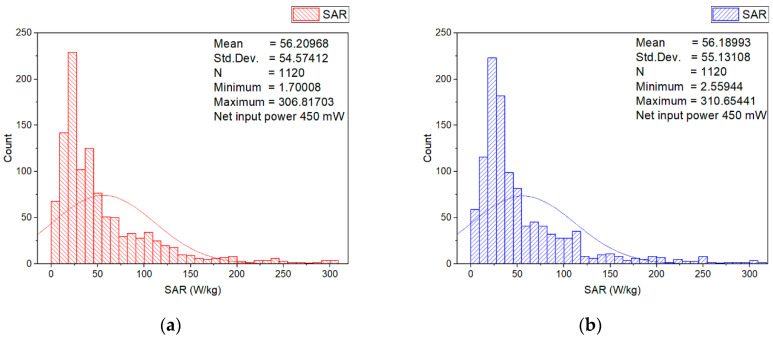

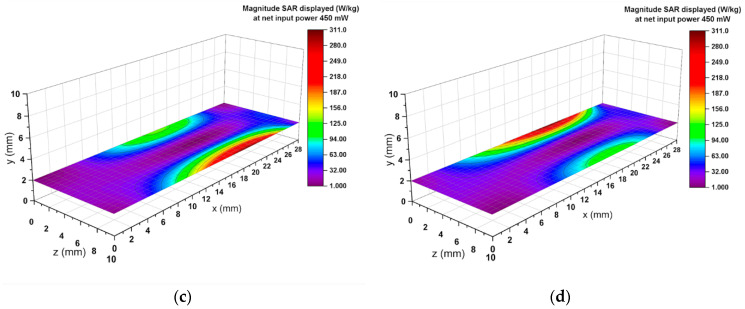
Histograms and descriptive statistics of SAR distribution for the erythrocyte suspensions in the layer with the highest SAR values at net input power of 450 mW: (**a**) left cuvette; (**b**) right cuvette; (**c**) left cuvette color-scale visualization of the SAR distribution; (**d**) right cuvette color-scale visualization of the SAR distribution.

**Figure 7:**
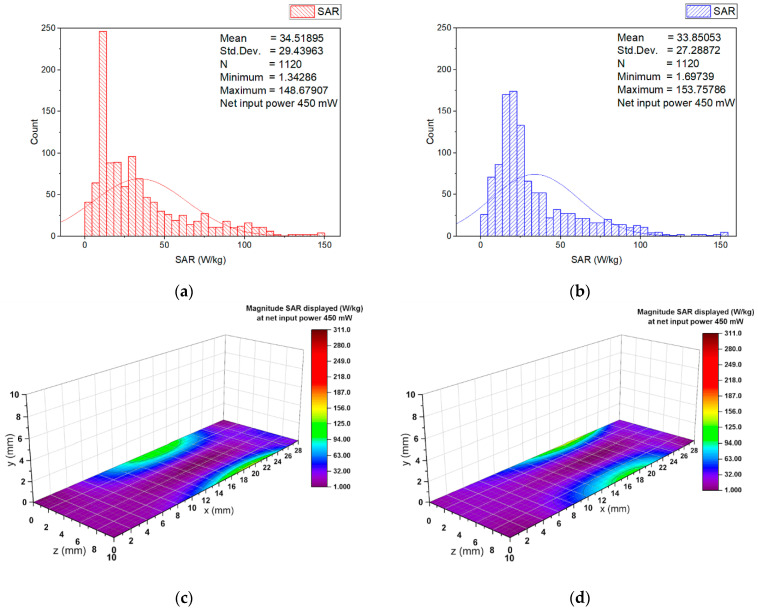
Histograms and descriptive statistics of SAR distribution at net input power of 450 mW for the erythrocyte suspensions in the layer at the bottom of (**a**) left cuvette; (**b**) right cuvette; (**c**) left cuvette color-scale visualization of the SAR distribution; (**d**) right cuvette color-scale visualization of the SAR distribution.

**Figure 8:**
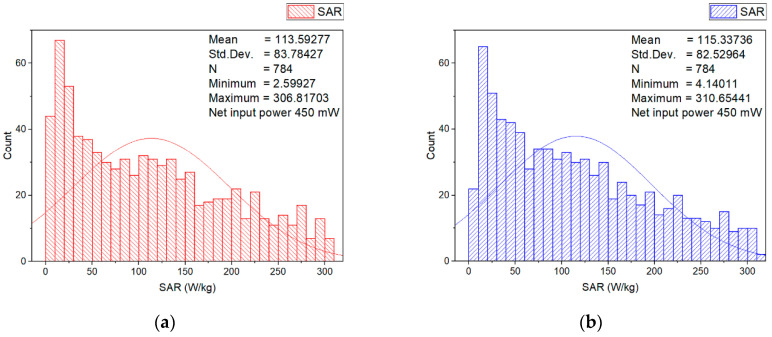

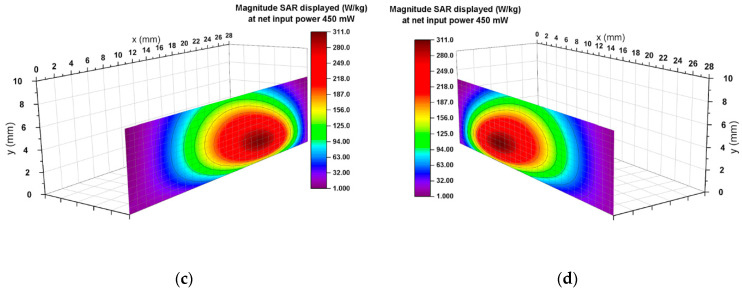
Histograms and descriptive statistics of SAR distribution at net input power of 450 mW for the erythrocyte suspensions in the cross-section of (**a**) left cuvette; (**b**) right cuvette; (**c**) left cuvette color-scale visualization of the SAR distribution; (**d**) right cuvette color-scale visualization of the SAR distribution.

In order to estimate the higher localized SAR levels induced from the wearable antenna in a small area from the numerical model of the erythrocyte suspension corresponding to a group of erythrocyte cells (for example, corresponding to a group of erythrocyte cells in a small blood vessel), the local SAR averaged over 0.125 mg in a cube with a volume of 0.125 mm^3^ of the numerical model of erythrocyte suspension at 2.41 GHz for two different input powers (6.3 mW and 450 mW) was investigated. The SAR distributions within the numerical model of erythrocyte suspension in the two cuvettes, representative histograms, and descriptive statistics at an input power of 450 mW, are shown in Figure 5, Figure 6, Figure 7 and Figure 8. 

Figure 5 shows the histograms and descriptive statistics of the local SAR (SAR averaged over 0.125 mg in a cube with a volume of 0.125 mm^3^) in the numerical model of erythrocyte suspension in the left (Figure 5a) and right (Figure 5b) cuvette, respectively. The SAR was scaled to 450 mW. As can be seen, in each of the two cuvettes, the SAR values were very similar, due to the symmetry in radiating elements of the wearable antenna geometry. From the results, it was also observed that the SAR values varied in the model of the erythrocyte suspension. Consequently, during the biological experiment, the SAR values could vary in individual groups of cells. The SAR values in 69% of the volume in the left and 70% in the right cuvette were lower than 50 W/kg. Moreover, a small part of the volume (under 1.51% in the left and 1.55% in the right cuvette) fell in the upper SAR range (SAR from 200 W/kg to 300 W/kg), which was undesirable for *in vitro* experiments as these indicated that significant thermal artifacts could occur [33] during long-term exposure. Hence, during the experimental investigation of the hemolysis of the human erythrocytes at long-term exposure with the fully textile-wearable antenna, the erythrocyte suspensions were stirred every hour in order to avoid heating (the appearance of so-called hot spots). To determine the location in which SAR values from 200 W/kg to 300 W/kg occurred, we calculated SAR in the longitudinal and cross-section of each layer (0.5 mm thick) of the numerical model of the erythrocyte suspension in the cuvettes. The results are shown in Figure 6, Figure 7 and Figure 8.

The highest local SAR was found 2 mm above the bottom of the cuvettes. Figure 6 displays the histograms, descriptive statistics, and color-scale visualization of the SAR distribution within the layer 2 mm above the bottom of the cuvettes. For both cuvettes, the peak SAR in the erythrocyte suspensions occurred in the locations in the antenna vicinity. Moreover, as can be seen from these results, 70% of SAR values in this layer were lower than 60 W/kg. 

The histograms, descriptive statistics, and color-scale visualization of the SAR distribution within the numerical model of the erythrocyte suspensions at the bottom of the cuvettes are shown in Figure 7. The peak local SAR in this layer was substantially (two times) lower than the maximum local SAR. For both cuvettes, the peak SAR in the numerical model of the erythrocyte suspension also occurred in the locations near the antenna vicinity. Moreover, as can be seen from these results, the SAR in 90% of the cell groups dropped below 60 W/kg. The results in Figure 6 and Figure 7 show that the highest SAR values occurred at locations in the wearable antenna vicinity. Hence, we calculated SAR in the numerical model of the erythrocyte suspension in the cross-section of both cuvettes at the location in the wearable antenna vicinity. The results are shown in Figure 8.

As shown in Figure 8, we observed a non-uniform SAR distribution in this layer. The SAR peaks were located at the center of the layer near each cuvette wall. The SAR distribution was non-uniform because the wearable antenna (source of EMF exposure) was at a location very close to erythrocyte suspension. As expected, when the source of the EMF was very close to the object, the electric and magnetic field components are not well defined, and the field strengths varied [17].

Similar SAR distributions were observed in the numerical model of the erythrocyte suspensions when the wearable antenna input power was 6.3 mW. A comparison of the maximum and minimum SAR values at the two input powers is given in Table 4. The maximum SAR value at an input power of 6.3 mW was 4.32492 W/kg. Moreover, a small group (only 4.5%) of RBCs fell in the area with maximum SAR. This meant that, during the biological experiments under realistic conditions, the SAR would not exceed the maximum permitted values established in [17,19].

### 3.3. Experimental Results on the Stability of Human Erythrocyte Membranes

In this subsection, we provide experimental results on the effects caused by electromagnetic fields emitted by the wearable antenna on the stability of human erythrocyte membranes. The erythrocytes were chosen as an object in this study because they are the main component of the human blood and constitute about 40–45% of blood volume [34]. As an indicator of disruption of the integrity of membranes (hemolysis), the level of the release of hemoglobin was investigated. Hemolysis may occur *in vitro* during collection and handling of the blood or in vivo due to some diseases such as hemolytic anemia or be provoked by various biotic and abiotic factors [35]. Accumulation of free hemoglobin in the body can cause many health issues, including heart disease or kidney stones [36]. Several previous studies reported changes in the stability of cell membranes [24,37] after irradiation of the erythrocyte suspensions with EMFs. To investigate the effects of EMF emitted by a textile wearable antenna on the stability of human erythrocyte membranes, we performed *in vitro* experiments at the variation of two important independent parameters: antenna input power and irradiation time.

#### 3.3.1. Effects of Weak EMFs Emitted by a Wearable Antenna on the Stability of the RBCs Membranes 

**Figure 9:**
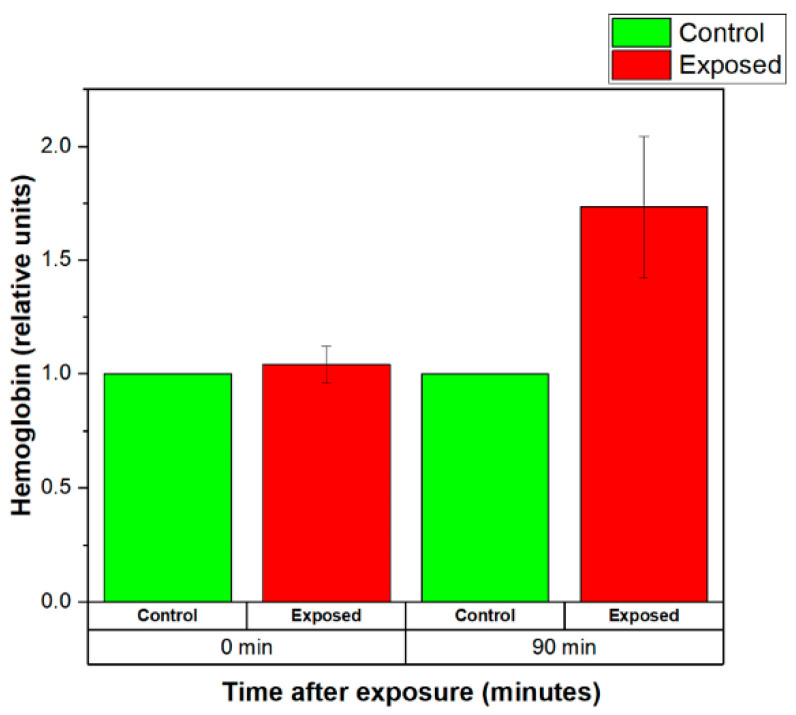
Normalized levels of the hemoglobin released by the control (sham-exposed) and exposed erythrocyte suspensions for 120 min to 2.41 GHz (WSA SAR = 0.4149 W/kg, psSAR1g = 0.5758 W/kg, wearable antenna input power 6.3 mW, Zigbee-like signal). The mean of 7 experiments ± SEM is shown.

The normalized levels of the hemoglobin released by the control (sham-exposed) and exposed for 120 min to 2.41 GHz (WSA SAR = 0.4149 W/kg, psSAR1g = 0.5758 W/kg, wearable antenna input power 6.3 mW, Zigbee-like signal) erythrocyte suspensions are shown in Figure 9. There was no effect of exposure to weak EMF emitted by the wearable antenna on the level of the released hemoglobin immediately after the end of the EMF exposure (Figure 9, 0 min). However, 90 min after EMF exposure, a difference in hemolysis of control and exposed groups was observed. The hemoglobin released by exposed RBCs increased to 1.7 ± 0.3.

**Figure 10:**
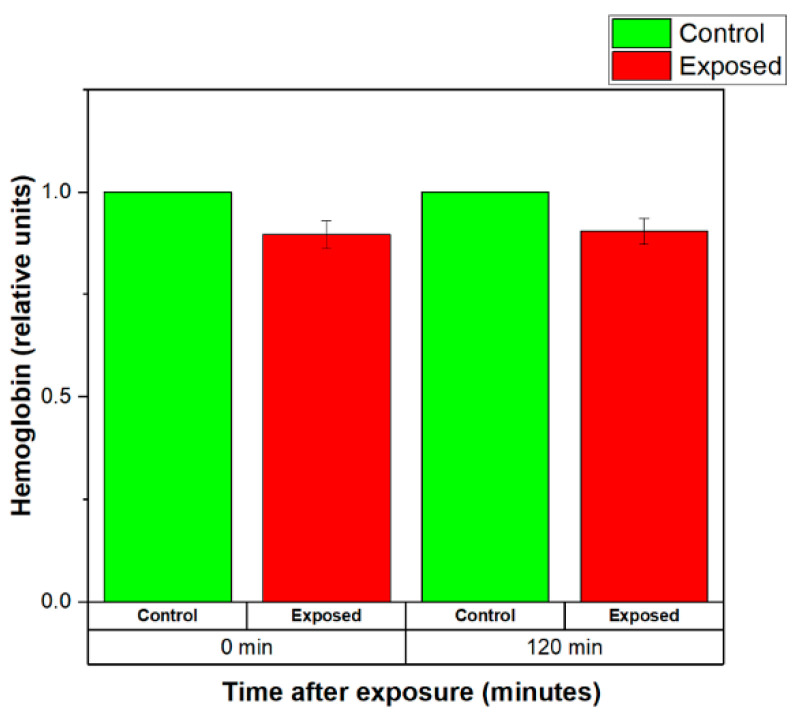
Normalized levels of the hemoglobin released by the control (sham-exposed) and exposed erythrocyte suspensions for 20 min to 2.41 GHz (WSA SAR = 0.4149 W/kg, psSAR1g = 0.5758 W/kg, wearable antenna input power 6.3 mW, Zigbee-like signal). The mean of 10 experiments ± SEM is shown.

The normalized levels of the hemoglobin released by the control (sham-exposed) and exposed for 20 min to 2.41 GHz (WSA SAR = 0.4149 W/kg, psSAR1g = 0.5758 W/kg, wearable antenna input power 6.3 mW, Zigbee-like signal) erythrocyte suspensions are shown in Figure 10. The results demonstrated that EMF-exposed erythrocyte suspensions released less hemoglobin than the control group both immediately after and 120 min after the end of the exposure (see Figure 10). The level of the hemoglobin released by exposed RBCs remained almost unchanged for 120 min after exposure. These results suggested that short-term (20 min) irradiation with weak EMF emitted from the wearable antenna stabilized the erythrocyte membranes.

#### 3.3.2. Effects of Strong EMFs Emitted by a Wearable Antenna on the Stability of the RBCs Membranes 

**Figure 11:**
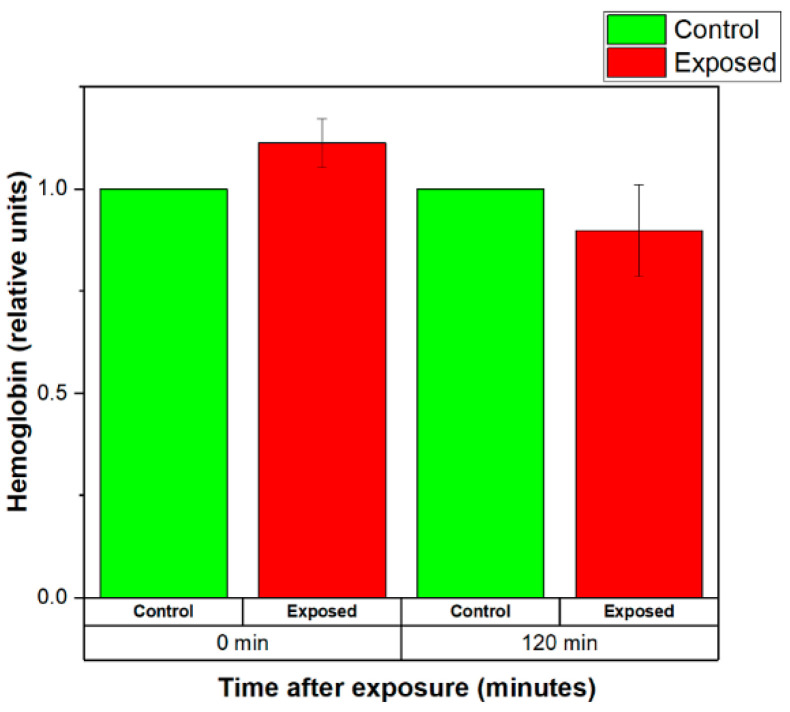
Normalized levels of the hemoglobin released by the control (sham-exposed) and exposed erythrocyte suspensions for 20 min to 2.41 GHz (WSA SAR = 29.6325 W/kg, psSAR1g = 41.13 W/kg, wearable antenna input power 450 mW, Zigbee-like signal). The mean of 4 experiments ± SEM is shown.

The normalized levels of the hemoglobin released by the control (sham-exposed) and exposed for 20 min to 2.41 GHz (WSA SAR = 29.6325 W/kg, psSAR1g = 41.13 W/kg, wearable antenna input power 450 mW, Zigbee-like signal) erythrocyte suspensions are shown in Figure 11. The results demonstrated that EMF-exposed erythrocyte suspensions released more hemoglobin than the control group immediately after the exposure (see Figure 11). Moreover, the level of the released hemoglobin by exposed RBCs decreased for 120 min after the end of the exposure. 

**Figure 12:**
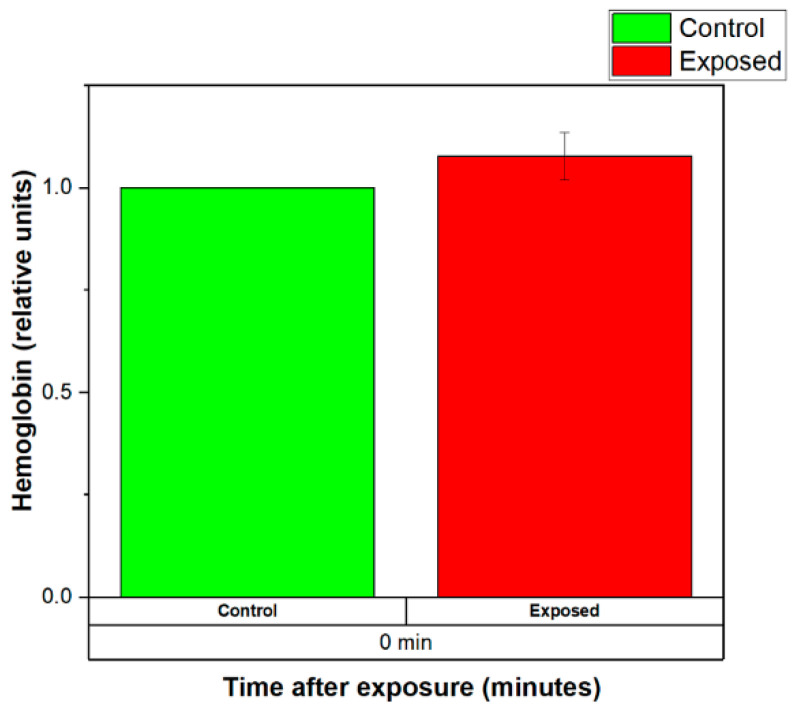
Normalized levels of the hemoglobin released by the control (sham-exposed) and exposed erythrocyte suspensions for 120 min to 2.41 GHz (WSA SAR = 29.6325 W/kg, psSAR1g = 41.13 W/kg, wearable antenna input power 450 mW, Zigbee-like signal). The mean of 4 experiments ± SEM is shown.

The normalized levels of the hemoglobin released by the control (sham-exposed) and exposed for 120 min to 2.41 GHz (WSA SAR = 29.6325 W/kg, psSAR1g = 41.13 W/kg, wearable antenna input power 450 mW, Zigbee-like signal) erythrocyte suspensions are shown in Figure 12. There were no significant increases in hemoglobin release at EMF-exposed RBCs.

#### 3.3.3. Comparison of the Effects Induced by EMF and Conventional Heat Exposure on the Stability of Human Erythrocyte Membranes

**Figure 13:**
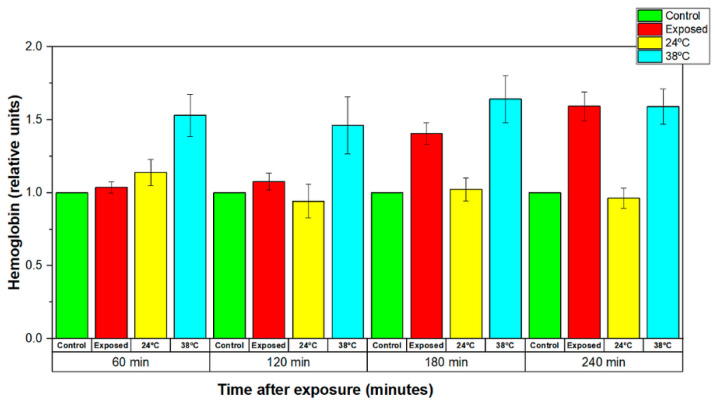
Normalized levels of the hemoglobin released by the control (sham-exposed), conventional heat exposed (38 °C) and EMF-exposed erythrocyte suspensions for 60, 120, 180, and 240 min to 2.41 GHz (WSA SAR = 29.6325 W/kg, psSAR1g = 41.13 W/kg, wearable antenna input power 450 mW, Zigbee-like signal). The mean of 4 experiments ± SEM is shown.

During the *in vitro* experiments, it was found that long-term EMF exposure under conservative exposure conditions led to an increase in the temperature of the erythrocyte suspensions. Additional experiments were performed in order to clarify the effects of temperature increase during the EMF exposure and those of conventional heating. The changes in the amount of released hemoglobin induced by 60, 120, 180, and 240 min irradiation with EMF under conservative exposure conditions (WSA SAR = 29.6325 W/kg, psSAR1g = 41.13 W/kg, wearable antenna input power 450 mW, Zigbee-like signal, 2.41 GHz) were compared with the changes in the amount of released hemoglobin induced by 60, 120, 180 and 240 min conventional heating (up to 38 °C) of the erythrocyte suspensions. The results demonstrated that both the EMF-exposed and conventionally heated samples released significantly more hemoglobin than the control groups and samples at room temperature (24 °C) after 180 min and 240 min exposure (see Figure 13). From the results, we were able to assume that the changes in the stability of erythrocyte membranes after 20 min and 120 min irradiation were mainly due to the effects of EMF emitted by the wearable antenna.

#### 3.3.4. Discussion

Results presented in this paper demonstrated that the short-term (20 min) irradiation of erythrocyte membranes in the reactive near-field of a wearable antenna at 6.3 mW input power had a stabilizing effect. At the same time, longer-term exposure (120 min) had a destabilizing effect on the erythrocyte membrane. The observed effects were a result of non-thermal specific mechanisms of interaction of the applied field.

In our previous experiments, we observed a statistically significant decrease in the quantity of the released hemoglobin during exposure of human erythrocytes to 900 MHz EMF (40% Ht, GSM900 EMF, 20 min) and during the first hour after the end of the treatment. The level of applied EM radiation was low enough to be considered non-thermal [37]. Moreover, data on the effects of EMF on hemolysis in available literature were contradictory. Several earlier studies [38,39] showed a significant increase in hemoglobin after exposure to 2.45 GHz EMF. The authors explained the effect of temporary membrane permeabilization rather than erythrocyte lysis. One reason for these differences was probably a difference in the experimental conditions of EMF exposure. Consequently, more investigations need to be carried out in order to understand the mechanisms of interaction between the reactive near-field of a wearable antenna and human erythrocyte membranes.

#### 3.3.5. Comparison

Finally, a comparison of the results and exposure conditions of these *in vitro* experiments with other similar *in vitro* experiments reported recently in the literature is given in Table 5 to highlight the novelty of the studies in this work.

As can be seen, current *in vitro* studies on RBCs [29,31,37,43] have not provided data for the correct estimation of the disruption of the RBCs membranes under real-life exposure conditions. In [29,31,37], SAR was not assessed. Although [40,41] provided data for average SAR, only high exposure levels were used to determine the change in the RBCs membranes and A172 cells. Moreover, only one *in vitro* experiment [42] applied SAR levels corresponding to real usage exposure conditions, and they did so using 1.8 GHz. Data on biological effects under real-life and conservative exposure conditions from wearable antennas or devices have not been reported. To overcome this gap, in this work, we investigated the possible biological responses of human erythrocyte membranes to electromagnetic fields from a fully textile wearable antenna at realistic (average SAR 0.4149 W/kg) and conservative (average SAR 29.6325 W/kg) exposure conditions at 2.41 GHz, Zigbee-like signal. Furthermore, results from a detailed SAR estimation, histograms, and descriptive statistics of the local SAR and SAR distributions in a numerical model of erythrocyte suspension—based on FDTD numerical simulations—were presented. This served to increase the quality of data on electromagnetic exposures applied during *in vitro* experiments and to assess potential adverse effects of electromagnetic exposure from wearable antennas.

## 4. Conclusions

This paper reported a detailed investigation of the SAR induced in erythrocyte suspensions from a fully textile wearable antenna at realistic (net input power 6.3 mW) and conservative (net input power 450 mW) conditions at 2.41 GHz. The examination of the SAR at the above conditions showed that the 1 g average SARs were 0.5758 W/kg (at a net input power of 6.3 mW) and 41.13 W/kg (at a net input power of 450 mW), respectively. Additionally, the local SAR, induced from the wearable antenna in a group of cells from the erythrocyte suspensions, was investigated. The SAR distributions within several longitudinal- and cross-sections of erythrocyte suspensions, representative histograms, and descriptive statistics were presented. Two new exposure systems were developed, and *in vitro* experiments were carried out. The obtained results from the *in vitro* experiments demonstrated that the short-term (20 min) irradiation of erythrocyte membranes in the reactive near-field of the wearable antenna at 6.3 mW input power had a stabilizing effect. Long-term exposure (120 min) to both investigated wearable antenna input powers had a destabilizing effect on the erythrocyte membrane.

## Figures and Tables

**Table 1:** Electromagnetic properties of the materials involved in the wearable antenna geometry erythrocyte suspension, plastic box, and flat phantom.

Materials	ε_r_′ ^1^	σ ^2^	Density ^3^
Wearable antenna substrate (polyester)	1.62	0.00118	1380
Erythrocyte suspension	58.20	2.59	1000
Plastic cuvette	2.2	0	880
Homogeneous flat phantom	40.805	2.33	1166

^1^ ε_r_′ real part of the complex permittivity. ^2^ σ—electrical conductivity, in S/m. ^3^ Density, in kg/m^3^.

**Table 2:** Comparison of the FDTD-computed antenna parameters at 2.41 GHz, in two scenarios: antenna in the free space, and antenna with two cuvettes filled with erythrocyte suspension.

Parameters	Antenna in Free Space	Antenna with Two Cuvettes
Impedance (Ohms)	89.52 + j16.47	49.71 + j5.29
VSWR	1.87	1.10
Dissipated power (% of net input power)	47.57	65.04
Radiation efficiency (%)	52.43	36.40
System efficiency (%)	47.59	36.31

**Table 3:** Peak 1 g spatial-average SAR and SAR averaged over the entire mass of the cell suspension produced in the numerical model of erythrocyte suspension at two input powers of the wearable antenna, at 2.41 GHz.

SAR ^1^	Input Power 6.3 mW	Input Power 450 mW
psSAR_1g_	0.5758	41.13
WSA SAR	0.4149	29.6325

^1^ SAR in W/kg.

**Table 4:** Comparison of the maximum and minimum SAR values at two input powers of the wearable antenna, at 2.41 GHz.

SAR ^1^	Input Power 6.3 mW	Input Power 450 mW
Minimum SAR	0.0187	1.3358
Maximum SAR	4.3492	310.6544

^1^ SAR in W/kg.

**Table 5:** Comparison of *in vitro* studies on biological effects from EMFs.

Reference	Cell Type	Exposure Conditions	Duration	SAR ^1^	Results
This work	Red blood cells—RBCs (Human erythrocytes)	2.41 GHz Zigbee signal Wearable antenna 6.3 mW input power	20 min	0.4149	RBCs membrane stabilization(decreased quantity of released hemoglobin (Hb))
This work	RBCs (Human erythrocytes)	2.41 GHz Zigbee signal Wearable antenna 6.3 mW input power	120 min	0.4149	RBCs membrane destabilization (increased level of released Hb 90 min after the exposure)
This work	RBCs (Human erythrocytes)	2.41 GHz Zigbee signal Wearable antenna 450 mW input power	20 min	29.6325	Increased level of released Hb immediately after the end of the exposure, relaxation to control levels 120 min later
This work	RBCs (Human erythrocytes)	2.41 GHz Zigbee signal Wearable antenna 450 mW input power	120 min	29.6325	Increased level of released Hb immediately after the end of the exposure
[29]	RBCs (Human erythrocytes)	2.45 GHz Continuous-wave (CW) irradiation chamber incident power density 0.84 mW/cm^2^	3600 min	NA	Hemolysis degree increases quasi-linearly with the exposure time
[29]	RBCs (Human erythrocytes)	2.45 GHz CW irradiation chamber incident power density 5 mW/cm^2^	3600 min	NA	Osmotic resistance of exposed RBCs increases in time, while the osmotic resistance of the controls is constant
[31]	RBCs (Human erythrocytes)	0.902 GHz GSM signal Nokia 6150 is programmed to emit at 902 MHz with 2 W erythrocyte suspensions in the main lob of the antenna radiation pattern	20 min	NA	Statistically significant decrease in the hemoglobin level in irradiated suspensions
[37]	RBCs (Human erythrocytes)	0.902 GHz GSM signal Nokia 6150 is programmed to emit 2 W erythrocyte suspensions at 2 cm distance from phone helical antenna	20 min	NA	Statistically significant decrease in the hemoglobin level in irradiated suspensions
[40]	RBCs (New Zealand white rabbit erythrocytes)	18 GHz CW Vari-Wave Model LT 1500	1 min	3000	Disturbance of the RBCs membranes
[41]	A172 cells (Human malignant glioblastoma)	2.45 GHz CW rectangular waveguide (TE_10_ mode)	120 min	5–20	No statistically significant changes in HSP 70, HSP27, and P-HSP27 in A172 cells
[41]	A172 cells (Human malignant glioblastoma)	2.45 GHz CW rectangular waveguide (TE_10_ mode)	120 min	50	A statistically significant increase in HSP 70 and p-HSP27 in A172 cells
[42]	Human peripheral blood lymphocytes	1.95 GHz UMTS signal exposure chamber	1200 min	0.3	No significant increase in the incidence of micronuclei compared with those of unexposed controls
[43]	Human epidermal cells—simulation model	1.8 GHz CW Wearable antenna—simulation model Gain 4.69 dBi Input power 290 mW	NA	1.125	Temperature increaseof 0.17 °C

^1^ The SAR averaged over the entire mass of the cell suspensions in W/kg.
